# Supplementary material for: First Identification and Phylogenetic Analysis of Porcine Circovirus Type 4 in Fur Animals in Hebei, China
Source: Animals (Basel). 2022 Nov 28;12(23):3325. doi: 10.3390/ani12233325 (PMC9737481; doi:10.3390/ani12233325)
Supplement: Supplementary file 1 [file animals-12-03325-s001.zip › animals-2031068-supplementary.pdf]

Table S1. 137 clinical samples of fur animals collected from 110 fur animal farms in Hebei Province, China.

Number	Farm	Coordinate (East longititude, north latitude)	Species of fur animal	Organ	Collected date	Detecting results		
						PCV4	PCV3	PCV2
1	1	119.251,39.814	Raccoon dog	Spleen	2015.3.2	-	-	-
2	2	119.315,39.833	Raccoon dog	Spleen	2015.3.23	-	-	+
3	3	119.019,39.775	Raccoon dog	Lung	2015.3.27	-	-	-
4	4	119.420,40.22	Raccoon dog	Spleen	2015.10.1	+	-	+
5	5	119.092,40.162	Raccoon dog	Spleen	2015.10.2	-	-	+
6	6	118.863,39.479	Raccoon dog	Lung	2015.10.31	-	-	-
7	7	118.789,39.463	Mink	Serum	2015.11.1	-	-	+
8	8	119.314,39.833	Raccoon dog	Spleen	2015.4.28	-	-	-
9	9	119.207,39.816	Raccoon dog	Lung	2015.6.24	-	+	+
10	10	119.177,39.815	Fox	Spleen	2015.6.27	-	+	-
11	11	119.013,39.705	Raccoon dog	Serum	2015.7.13	+	+	+
12	12	119.210,40.229	Mink	Serum	2015.7.13	-	-	+
13	13	119.127,40.145	Raccoon dog	Lung	2015.7.16	-	-	-
14	14	119.255,39.459	Fox	Liver	2015.7.28	-	-	+
15	15	119.222,39.425	Raccoon dog	Liver	2015.7.29	+	+	+
16	16		Raccoon dog	Spleen	2015.7.14	-	-	-
17	16	119.950,40.252	Raccoon dog	Spleen	2017.9.25	-	-	+
18	16		Raccoon dog	Spleen	2019.7.14	-	-	-
19	17	119.064,40.226	Raccoon dog	Serum	2015.8.15	-	-	+
20	18	119.0180,40.162	Raccoon dog	Serum	2015.8.16	-	+	-
21	19	119.222,39.425	Raccoon dog	Spleen	2015.8.5	-	-	-
22	20	119.264,39.829	Mink	Spleen	2015.8.5	+	-	+

23	21	119.299,39.857	Raccoon dog	Lung	2015.9.2	-	-	+
24	22	119.209,39.78	Raccoon dog	Lung	2015.9.22	-	-	-
25	23	119.314,39.833	Raccoon dog	Brain	2017.5.15	-	-	-
26	24	119.069,39.744	Raccoon dog	Lung	2017.6.27	-	-	+
27	25	118.934,40.116	Raccoon dog	Small intestine	2017.6.29	-	+	+
28	26	118.901,40.021	Raccoon dog	Small intestine	2017.1.13	-	-	+
29	27	119.050,39.746	Raccoon dog	Lung	2017.10.23	-	-	+
30	27		Raccoon dog	Lung	2019.7.31	-	-	-
31	28	118.934,40.116	Raccoon dog	Spleen	2017.2.19	-	-	+
32	29	118.974,40.205	Raccoon dog	Lung	2017.2.26	-	+	+
33	29		Raccoon dog	Lung	2019.2.26	-	+	+
34	30	119.073,39.705	Mink	Serum	2017.2.9	+	+	+
35	31	119.103,39.689	Raccoon dog	Lung	2017.6.22	-	-	+
36	32	118.844,39.465	Raccoon dog	Brain	2017.6.27	-	-	+
37	33	118.836,39.401	Fox	Lung	2017.7.19	-	+	+
38	34	119.131,39.659	Raccoon dog	Liver	2018.12.23	-	+	-
39	35	119.193,39.660	Raccoon dog	Lung	2018.6.15	-	-	+
40	36	119.203,39.640	Raccoon dog	Lung	2018.6.22	+	+	-
41	37	119.220,39.636	Raccoon dog	Lymph node	2018.6.24	-	-	-
42	38		Fox	Spleen	2018.6.25	-	-	-
43	38	119.223,39.585	Raccoon dog	Spleen	2018.6.25	-	-	-
44	38		Raccoon dog	Spleen	2018.6.25	+	+	+
45	39	119.245,39.604	Fox	Lung	2018.6.26	+	+	+
46	40	119.250,39.726	Raccoon dog	Spleen	2018.6.29	-	+	-
47	41	119.220,39.763	Raccoon dog	Lung	2018.6.29	-	+	-
48	42	119.196,39.771	Raccoon dog	Lung	2018.7.11	-	+	+
49	43	119.179,39.789	Raccoon dog	Lymph node	2018.7.16	-	-	-

50	44	119.224,39.830	Raccoon dog	Lung	2018.7.17	-	+	+
51	45	119.295,39.858	Raccoon dog	Lung	2018.7.25	-	+	-
52	46	119.315,39.832	Raccoon dog	Lung	2018.7.27	-	-	+
53	47	119.250,39.726	Raccoon dog	Lymph node	2018.7.9	+	+	-
54	48	118.844,39.465	Raccoon dog	Lung	2018.8.19	-	+	-
55	49	119.315,39.832	Raccoon dog	Lung	2018.8.31	+	-	-
56	50	119.363,39.863	Raccoon dog	Lung	2018.9.2	-	+	-
57	51	119.294,39.92	Raccoon dog	Lung	2019.3.19	-	+	-
58	52	119.444,39.887	Raccoon dog	Serum	2019.3.19	+	+	-
59	52		Raccoon dog	Serum	2019.3.19	+	+	-
60	53	119.250,39.726	Raccoon dog	Lung	2019.7.10	-	-	+
61	54	119.374,39.877	Raccoon dog	Lymph node	2019.1.16	-	-	-
62	55	119.442,39.887	Raccoon dog	Lung	2019.10.11	-	-	+
63	56	119.386,39.981	Raccoon dog	Spleen	2019.10.2	-	-	+
64	57	119.476,39.989	Raccoon dog	Lung	2019.10.31	-	-	-
65	58	119.374,39.877	Raccoon dog	Spleen	2019.10.4	-	-	+
66	59	119.423,39.976	Raccoon dog	Lung	2019.10.9	-	-	+
67	60	119.220,39.763	Raccoon dog	Lymph node	2019.5.27	-	-	-
68	61	119.301,40.124	Raccoon dog	Spleen	2019.6.12	-	-	-
69	62	119.295,40.11	Raccoon dog	Spleen	2019.6.12	-	+	+
70	63	119.231,40.104	Raccoon dog	Lung	2019.6.22	-	-	-
71	64	119.220,40.07	Raccoon dog	Lung	2019.6.24	-	-	-
72	65	119.248,40.120	Fox	Spleen	2019.6.3	-	-	-
73	66	119.295,40.11	Raccoon dog	Lung	2019.6.7	-	-	-
74	67	119.179,39.789	Raccoon dog	Spleen	2019.7.1	-	-	-
75	68	119.374,39.877	Raccoon dog	Serum	2019.7.15	-	-	-
76	69	119.295,40.11	Raccoon dog	Lymph node	2019.7.20	-	-	-

77	69		Raccoon dog	Spleen	2019.7.20	-	-	-
78	70	119.206,40.107	Raccoon dog	Spleen	2019.7.20	-	+	-
79	71	119.442,39.887	Raccoon dog	Spleen	2019.7.4	-	-	+
80	72	119.157,40.073	Raccoon dog	Kidney	2019.8.24	-	-	-
81	73	119.121,40.284	Raccoon dog	Lung	2019.8.25	-	-	-
82	74	119.097,40.316	Raccoon dog	Lung	2019.8.7	+	-	-
83	75	119.135,40.335	Raccoon dog	Spleen	2019.8.8	-	+	-
84	76	119.220,39.763	Raccoon dog	Lung	2019.9.03	-	+	-
85	77	119.120,40.359	Raccoon dog	Spleen	2019.9.17	-	-	+
86	78	119.105,40.364	Fox	Lung	2019.9.2	-	-	+
87	79	119.096,40.472	Raccoon dog	Lymph node	2019.9.30	-	-	+
88	80	119.281,40.146	Raccoon dog	Serum	2019.9.12	-	-	+
89	80		Raccoon dog	Serum	2019.9.12	-	-	+
90	81	119.248,40.120	Raccoon dog	Spleen	2020.9.29	-	+	-
91	81		Raccoon dog	Spleen	2020.9.29	-	+	-
92	82	119.248,40.109	Raccoon dog	Lung	2019.5.23	-	-	-
93	82		Raccoon dog	Kidney	2019.5.23	-	-	-
94	82		Raccoon dog	Lung	2019.6.3	-	+	+
95	82		Raccoon dog	Spleen	2019.6.13	-	-	-
96	82		Raccoon dog	Spleen	2019.6.18	+	-	-
97	82		Raccoon dog	Spleen	2019.4.3	+	-	-
98	82		Raccoon dog	Spleen	2019.4.5	-	-	+
99	82		Raccoon dog	Liver	2019.4.16	-	-	-
100	82		Raccoon dog	Kidney	2019.4.18	-	-	-
101	82		Raccoon dog	Lung	2019.4.18	-	-	-
102	83	119.053,40.323	Mink	Liver	2017.9.13	+	-	-
103	83	119.067,40.341	Raccoon dog	Spleen	2017.9.13	+	-	+

104	84	119.067,40.341	Raccoon dog	Kidney	2017.11.11	+	-	-
105	85	119.173,40.121	Raccoon dog	Lung	2017.10.27	-	+	-
106	86	119.126,40.273	Mink	Spleen	2017.8.21	-	-	-
107	87	119.147,40.508	Mink	Spleen	2017.6.23	-	-	-
108	88	119.177,40.116	Mink	Lung	2017.12.9	-	+	+
109	88		Mink	Kidney	2017.12.9	+	-	-
110	88		Mink	Kidney	2017.12.9	+	-	+
111	88		Mink	Kidney	2017.12.9	+	+	+
112	89	119.295,40.11	Raccoon dog	Lung	2017.10.14	+	-	+
113	90	119.121,40.284	Fox	Kidney	2017.10.2	+	-	-
114	91	119.126,40.273	Raccoon dog	Lung	2017.10.27	+	-	-
115	92	119.181,40.515	Raccoon dog	Spleen	2016.6.29	+	-	-
116	93	119.203,40.498	Raccoon dog	Kidney	2017.7.1	+	+	-
117	94	119.270,40.575	Fox	Brain	2017.11.17	-	-	-
118	94		Fox	Lung	2017.11.17	-	-	+
119	95	119.720,40.217	Fox	Serum	2017.10.1	-	-	-
120	96	119.586,40.244	Raccoon dog	Spleen	2018.1.17	-	-	-
121	97	119.468,40.361	Fox	Liver	2017.11.17	-	-	-
122	97		Fox	Liver	2017.11.17	-	-	-
123	98	119.468,40.361	Raccoon dog	Spleen	2017.7.12	-	-	+
124	99	118.850,40.547	Fox	Spleen	2017.8.21	-	-	+
125	100	119.126,40.273	Raccoon dog	Spleen	2017.6.27	-	-	-
126	101	118.757,40.177	Fox	Liver	2017.11.11	-	-	-
127	102	119.222,39.830	Raccoon dog	Liver	2017.9.26	-	+	-
128	103	118.700,40.123	Mink	Lung	2017.12.16	-	-	-
129	103		Mink	Lung	2017.12.16	+	+	+
130	103		Fox	Lung	2017.12.16	+	-	-

131	104	118.922,40.219	Raccoon dog	Lung	2018.1.1	+	-	+
132	105	119.152,39.627	Raccoon dog	Lung	2018.1.23	+	-	-
133	106	118.978,39.785	Raccoon dog	Spleen	2017.6.28	-	-	-
134	107	118.880,39.801	Raccoon dog	Small intestine	2017.9.1	+	+	-
135	108	118.798,39.824	Raccoon dog	Lung	2018.1.9	-	-	+
136	109	118.939,39.955	Raccoon dog	Small intestine	2017.11.11	-	-	-
137	110	118.996,39.587	Raccoon dog	Spleen	2017.7.5	+	+	+

Table S2. Information of 86 representative isolates studied in this research.

Strain	Accession no.	Genotype	Strain	Accession no.	Genotype
PCV1	AY193712.1	PCV1	CN/NM1/2017	MT882410.1	PCV4
BJ-1	FJ475129.1	PCV1	JSYZ1901-2	MT769268.1	PCV4
Hun	KJ408799.1	PCV1	HNU-AHG1-2019	NC055580.1	PCV4
LV34	MN508363.1	PCV1	E115	MT882344.1	PCV4
XFD-Beijing	KC447455.1	PCV1	Henan-LY1-2019	MT015686.1	PCV4
HLJ1502	KY940535.1	PCV2	HNU-AHG1-2019	MK986820.1	PCV4
France	AF055394.1	PCV2	KU-02010	MW712668.1	PCV4
SD6	DQ218421.1	PCV2	JXSC-2021	MW988109.1	PCV4
DK1980PMWSfree	EU148503.1	PCV2	JXWY-2021	MW988108.1	PCV4
TJ	AY181946.1	PCV2	Hebei-AP1-2019	MW084633.1	PCV4
BJ0901b	GU001710.1	PCV2	HN-ZK-201707	MW600960.1	PCV4
GX0601	EF524532.1	PCV2	HN-XX-201601	MW600959.1	PCV4
Rac-hb1	MH373555.1	PCV2	HN-ZMD-201212	MW600958.1	PCV4
Rac-hb2	MH373556.1	PCV2	HN-ZK-201601	MW600957.1	PCV4
Rac-hb3	MH373557.1	PCV2	HN-ZK-201512	MW600956.1	PCV4
Rac-hb4	MH373558.1	PCV2	HN-ZZ-201603	MW600955.1	PCV4
Rac-hb5	MH373559.1	PCV2	KF-02-2019	MT193105.1	PCV4
Rac-hb6	MH373560.1	PCV2	KF-01-2019	MT193106.1	PCV4
FoxHB1	MH373552.1	PCV2	HN-LY-202005	MW538943.1	PCV4
FoxHB2	MH373553.1	PCV2	HN-LY-202006	MW600947.1	PCV4
FoxHB3	MH373554.1	PCV2	HN-LY-202007	MW600948.1	PCV4
Pig-hb1	MK305871.1	PCV2	HN-SMX-202011	MW600949.1	PCV4
Pig-hb2	MK305872.1	PCV2	HN-XX-201811	MW600950.1	PCV4
Pig-hb3	MK305873.1	PCV2	HN-KF-201812	MW600951.1	PCV4
Pig-hb4	MK305874.1	PCV2	HN-HB-201704	MW600952.1	PCV4
China/GD2016	KY418606.1	PCV3	HN-XX-201212	MW600953.1	PCV4
NWHEB21	MG564174.1	PCV3	HN-LY-201702	MW600954.1	PCV4
GDBL1	MF405272.1	PCV3	NG13	GQ404856.1	circular virus
Jiangxi-3	MF589106.1	PCV3	Canine circovirus 214	JQ821392.1	Canine circovirus

CN FJ-1	KY753912.1	PCV3	Canine circovirus C85	MK944080.1	Canine circovirus
CHN/GD2016	KY421347.1	PCV3	Mulard duck circovirus DuCV	AY228555.1	Mulard duck circovirus
Guangdong-HY1	MF589102.1	PCV3	Duck circovirus-FJZZ302	GQ423747.1	Duck circovirus
MO2015	KX778720.1	PCV3	Fox circovirus-55590	KP941114.1	Fox circovirus
Henan-13	KY075988.1	PCV3	Fox circovirus-VS7100003	KP260926.1	Fox circovirus
KU-1608	KY996344.1	PCV3	Zebra finch circovirus 32469	KU641384.1	Zebra finch circovirus
GX2020/FCG49	MT311854.1	PCV4	Finch circovirus	DQ845075.1	Finch circovirus
GX2020/GL69	MT311853.1	PCV4	Goose circovirus TW9-2001	AF536939.1	Goose circovirus
GX2020/NN88	MT311852.1	PCV4	Goose circovirus-JX1	GU320569.1	Goose circovirus
PCV4-LY2017	MW759029.1	PCV4	Canary circovirus	AJ301633.1	Canary circovirus
PCV4-HB2017	MW759028.1	PCV4	Mink circovirus SD16	MG001455.1	Mink circovirus
PCV4-YY2019	MW759027.1	PCV4	Mink circovirus MiCV-DL13	NC023885.1	Mink circovirus
PCV4-LY2020	MW759026.1	PCV4	Bat associated circovirus 1 XOR	NC038385.1	Bat circovirus
FJ-PCV4	MT721742.1	PCV4	Bat circovirus-Daqing3	KX756994.1	Bat circovirus
